# Clinically Meaningful Improvements in LUTS/BPH Severity in Men Treated with Silodosin Plus Hexanic Extract of Serenoa Repens or Silodosin Alone

**DOI:** 10.1038/s41598-017-15435-0

**Published:** 2017-11-09

**Authors:** Luca Boeri, Paolo Capogrosso, Eugenio Ventimiglia, Walter Cazzaniga, Filippo Pederzoli, Donatella Moretti, Federico Dehò, Emanuele Montanari, Francesco Montorsi, Andrea Salonia

**Affiliations:** 1 Division of Experimental Oncology/Unit of Urology; URI; IRCCS Ospedale San Raffaele, Milan, 20132 Italy; 2 IRCCS Fondazione Ca’ Granda–Ospedale Maggiore Policlinico; Department of Urology, Milan, 20122 Italy; 3 University Vita-Salute San Raffaele, Milan, 20132 Italy

## Abstract

To assess the rate and predictors of clinically meaningful improvements (CMI) in patients with lower urinary tract symptoms (LUTS) treated with either silodosin (SIL) alone or with a combination of SIL+ serenoa repens (Ser) hexanic lipidosterolic extract for ≥12 months. Data from 186 patients were collected. Patients completed the International Prostatic Symptoms Score (IPSS) at baseline and at follow-up assessment. Descriptive statistics and logistic regression models tested rates and predictors of CMI. Two CMI were assessed: 1) >3 points improvement in total IPSS from baseline to end (CMI#1); 2) >25% IPSS improvement from baseline to end (CMI#2). Overall, 93 (50%) patients were treated with SIL and SIL+ Ser, respectively. At a mean 13.5-mos follow-up [range: 12–20], mean IPSS scores were significantly lower in patients treated with SIL + Ser compared to those after SIL (p = 0.002). SIL + Ser patients more frequently achieved CMI#1 (69.9% vs. 30.1%, p = 0.001) and CMI#2 (68.8% vs. 31.2%, p < 0.001) compared SIL men. At multivariable analyses, younger age, IPSS severity and SIL + Ser (all p < 0.03) were independent predictors of CMI#1 and CMI#2. In conclusion, SIL + Ser therapy was more effective than SIL alone in improving IPSS scores in men with LUTS. SIL + Ser treatment led to CMIs in up to seven out of ten men.

## Introduction

Benign prostatic hyperplasia (BPH) is the most common cause of lower urinary tract symptoms (LUTS) in men and is strongly associated with ageing^1^. The pathogenesis of LUTS/BPH is not fully understood^1^, but a growing body of literature suggests that chronic prostatic inflammation represents a crucial component in the pathogenesis of BPH per se^2^ and is clearly associated with the severity of the disease^3,4^.

To date, alpha-blockers are widely considered the first-line treatment for men with moderate-to-severe LUTS/BPH^1,5^. Phytotherapeutic agents are currently used with the aim of relieving LUTS severity while avoiding the possible adverse events of the typical pharmacological agent for BPH. In this context, because they are a heterogeneous group and may contain different concentrations of the active ingredients, current published findings concerning their effectiveness in terms of LUTS/BPH relief have to be interpreted with caution^6^. To this aim, the European Association of Urology (EAU) Guidelines, for instance, did not report any specific recommendations on phytotherapy for the treatment of male LUTS^1^.

The hexanic lipidosterolic extract of serenoa repens (Ser) has been used worldwide for more than three decades to treat LUTS/BPH and its use has increased significantly in recent years^7^. The beneficial pharmacological effects of Ser have been associated to its anti-androgenic, anti-proliferative, pro-apoptotic and anti-edemic activity^7,8^. Moreover, Ser has a major anti-inflammatory effect on prostatic tissue and has been suggested to inhibit the 5α-reductase enzymes^8–10^. Previous studies have shown the benefit of Ser, used as a monotherapy, in reducing prostate size and urinary frequency, as well as in improving urine flow-rate and reducing nocturia^11^. Conversely, other studies have not supported these claims^6,12^. Of clinical relevance, only a few studies with not univocal findings have evaluated the efficacy of implementing Ser together with alpha-blockers (any type) to relieve LUTS/BPH severity^13,14^.

To the best of our knowledge, no studies have investigated the clinically meaningful improvements (CMI) in LUTS\BPH severity in patients treated with the combination of Ser and alpha-blockers as compared with the use of any alpha-blocker as a monotherapy. To this aim, we performed a cross-sectional study assessing rates of and predictors of CMI in LUTS\BPH patients treated with either silodosin 8 mg (SIL) alone or a combination therapy of SIL + Ser using validated questionnaires in a real-life setting.

## Results

The initial cohort of patients included 190 men equally distributed between groups. After matching, 93 patients were eventually considered for the study in the SIL alone and in the SIL + Ser groups at follow-up analysis, respectively.

Groups did not different with regard to demographic or clinical baseline parameters (Table 1). Mean follow-up assessment was performed after 13.5 months (range 12–20).

**Table 1:** Baseline characteristics and descriptive statistics of participants (No. = 186; mean (SD), [range]).

	**Overall**	**SIL**	**SIL** + **Ser**	p-value (F)*
No. of patients (%)	186 (100)	93 (50.0)	93 (50.0)	0.12 (2.5)
Age (years)	56.6 (11.8)	57.9 (11.3)	55.3 (12.2)	
	[41–84]	[42–84]	[41–81]	
BMI [kg/m^2^]	25.4 (3.2)	25.3 (2.8)	25.5 (3.6)	0.69 (0.15)
	[15.7–42.2]	[19.1–35.1]	[15.7–42.2]	
CCI	0.26 (0.6)	0.29 (0.6)	0.23 (0.5)	0.46 (0.54)
	[0–3]	[0–3]	[0–2]	
CCI categorized [No. (%)]				0.25 (*X*_2_ = 1.3)
0	152 (81.7)	73 (78.5)	79 (84.9)	
≥1	34 (18.3)	20 (21.5)	14 (15.1)	
PSA (ng/ml)	2.0 (1.2)	2.1 (1.1)	1.8 (1.2)	0.21 (1.54)
	[0.3–4.0]	[0.3–4.0]	[0.2–4.0]	
Prostate Volume (ml)	50.2 (21.0)	52.9 (20.6)	47.5 (21.3)	0.19 (1.72)
	[18–90]	[20–90]	[18–90]	
Flow Max (ml/sec)	10.7 (3.4)	10.9 (3.3)	10.3 (3.3)	0.32 (0.97)
	[4.0–15.0]	[4.0–15.0]	[4.0–15.0]	
PVR (ml)	51.1 (46.9)	54.8 (45.7)	46.3 (48.5)	0.32 (0.98)
	[0–150]	[0–150]	[0–150]	
Total IPSS score	17.7 (5.3)	17.3 (5.5)	18.2 (5.1)	0.22 (1.51)
	[12–33]	[12–33]	[12–31]	
IPSS - categorized [No. (%)]				0.87 (*X*_2_ = 0.02)
Moderate	123 (66.1)	62 (66.7)	61 (65.6)	
Severe	63 (33.9)	31 (33.3)	32 (34.4)	
Storage Symptoms	7.6 (3.1)	7.5 (3.1)	7.7 (3.1)	0.55 (0.35)
	[1–16]	[1–14]	[1–16]	
Voiding Symptoms	9.9 (3.7)	9.5 (3.8)	10.5 (3.6)	0.06 (3.47)
	[0–19]	[2–19]	[0–18]	
IPSS–QoL	3.3 (1.5)	3.2 (1.6)	3.5 (1.4)	0.19 (1.71)
	[0–6]	[0–6]	[0–6]	
IIEF– EF	17.6 (6.3)	18.2 (6.3)	17.1 (6.4)	0.52 (0.41)
	[2–25]	[2–25]	[2–25]	
IIEF - EF categorized [No. (%)]				0.56 (*X*_2_ = 1.14)
No ED	49 (26.2)	27 (28.6)	23 (24.2)	
Mild	64 (34.4)	36 (39.3)	28 (30.3)	
Moderate to Severe	73 (39.3)	30 (32.1)	42 (45.5)	
Follow up (months)	13.5 (1.7)	13.7 (1.6)	13.4 (1.7)	0.51 (0.44)
	[12–20]	[12–20]	[12–18]	

Keys: SIL = Silodosin; SIL + Ser = Silodosin + hexanic extract of serenoa repens; BMI = body mass index; CCI = Charlson Comorbidity Index; PSA = Prostate Specific Antigen; PVR = Postvoiding residual volume; IPSS = International Prostatic Index Score; QoL = Quality of Life; IIEF–EF = International Index of Erectile Function–Erectile Function domain; ED = Erectile dysfunction.

*P value according to chi-square test or analysis of variance (ANOVA), as indicated.

After a minimum period of 12 months of treatment, the difference between the International Prostate Symptom Score (IPSS) values at baseline and follow-up were significant in both groups (all p < 0.001), with a greater reduction observed for patients treated with SIL + Ser (−6.43 vs. −3.21) (Table 2). Overall, mean IPSS score was significantly lower for patients treated with SIL + Ser than for those with SIL monotherapy (p = 0.002). A greater proportion of patients treated with SIL monotherapy still had severe IPSS scores after treatment, compared to men treated with the combination therapy (19.5% vs. 11.8%, p = 0.014). Similarly, both storage and voiding symptoms were significantly lower in the SIL + Ser group, compared to SIL alone group (all p ≤ 0.04). In this context, as compared with baseline, a greater reduction in both storage (−2.41 vs. −0.79) and voiding symptoms (−3.8 vs. −1.6) was depicted in the SIL + Ser group as compared with the monotherapy group. Similar results were observed for the IPSS-QoL scores. The International Index of Erectile Function–Erectile Function domain (IIEF-EF) scores did not significantly and differentially improved at follow-up.

**Table 2:** Descriptive statistics of efficacy parameters at follow-up assessment and mean changes values from baseline (SD) (No. = 186).

	**SIL**	**SIL** + **Ser**	p-value (F)*
	**(No. = 93)**	**(No. = 93)**	
PSA (ng/ml)			0.17 (1.8)
Mean (SD)	2.1 (1.2)	1.8 (1.2)	
FU - baseline	−0.01 (0.3)	−0.34 (0.03)	
Prostate volume (ml)			0.21 (1.6)
Mean (SD)	52.3 (22.8)	46.9 (20.8)	
FU - baseline	−0.54 (1.3)	−0.6 (0.6)	
Flow Max (ml/sec)			0.15 (2.1)
Mean (SD)	13.2 (3.7)	14.3 (3.8)	
FU - baseline	2.31 (0.4)^§^	4.3 (0.5)^§^	
PVR (ml)			0.16 (1.9)
Mean (SD)	36.9 (41.5)	27.0 (36.3)	
FU - baseline	−17.8 (1.4)^§^	−19.3 (2.6)^§^	
Total IPSS score			0.002 (9.8)
Mean (SD)	14.5 (5.5)	11.8 (6.0)	
FU - baseline	−3.21 (0.6)^§^	−6.43 (0.6)^§^	
IPSS - categorized [No. (%)]			0.014 (*X*_2_ = 8.6)
Mild	8 (8.6)	22 (23.7)	
Moderate	67 (72.0)	60 (64.5)	
Severe	18 (19.5)	11 (11.8)	
Storage Symptoms			0.04 (8.6)
Mean (SD)	6.7 (3.1)	5.3 (3.2)	
FU - baseline	−0.79 (0.3)^**^	−2.42 (0.3)^§^	
Voiding Symptoms			0.044 (4.2)
Mean (SD)	7.8 (3.6)	6.7 (3.9)	
FU - baseline	−1.68 (0.44)^§^	−3.83 (0.4)^§^	
IPSS–QoL			0.024 (5.8)
Mean (SD)	3.1 (1.5)	2.5 (1.4)	
FU - baseline	−0.19 (0.2)	−0.97 (0.2)^§^	
IIEF– EF			0.58 (0.31)
Mean (SD)	18.3 (6.5)	17.3 (6.3)	
FU - baseline	0.1 (0.06)	0.2 (0.08)	
CMI#1 [No. (%)]	28 (30.1)	65 (69.9)	0.001 (*X*_*2*_ = 10.7)
CMI#2 [No. (%)]	29 (31.2)	64 (68.8)	< 0.001 (*X*_*2*_ = 18.2)

Keys: SIL = Silodosin; SIL + Ser = Silodosin + hexanic extract of serenoa repens; BMI = body mass index; CCI = Charlson Comorbidity Index; PSA = Prostate Specific Antigen; PVR = Postvoiding residual volume; IPSS = International Prostatic Index Score; QoL = Quality of Life; IIEF–EF = International Index of Erectile Function–Erectile Function domain; ED = Erectile dysfunction; CMI = Clinically meaningful improvements.

*P value according to chi-square test or analysis of variance (ANOVA), as indicated.

^§^p < 0.001; **p < 0.01.

Overall, SIL + Ser patients more frequently achieved CMI#1 (p < 0.001) and CMI#2 (p < 0.001) compared to men in the SIL alone group.

Of all, treatment-emergent adverse effects (TEAEs) were reported by 27 (29.0%) and 29 (31.2%) patients in the SIL and SIL + Ser group, respectively, with no significant differences between groups (Table 3). The most frequently reported adverse reactions were ejaculatory disorders followed by slight postural hypotension. None patient discontinued the therapies because of the side effects.

**Table 3:** Summary of adverse events in the two groups [No. (%)].

	**SIL**	**SIL** + **Ser**	
Overall	27 (29.0)	29 (31.2)	p = 0.25 (*X*_*2*_ = 1.7)
Headache	0 (0)	2 (2.2)	
Postural hypotension	7 (7.5)	5 (5.4)	
Decreased Libido	3 (3.2)	2 (2.2)	
Ejaculatory disorders	10 (10.7)	12 (12.8)	
Dry mouth	4 (4.4)	3 (3.2)	
Rhinitis	3 (3.2)	1 (1.1)	
Stomach upset	0 (0)	4 (4.4)	

Keys: SIL = Silodosin; SIL + Ser = Silodosin + hexanic extract of serenoa repens.

Table 4 reports univariable (UVA) and multivariable (MVA) analyses assessing the association between predictors and CMI in terms of LUTS severity. At UVA, younger age, baseline IPSS and the combination of SIL + Ser were significantly associated with CMI#1 and CMI#2 (all p < 0.02). At MVA, younger age (OR 0.97, p = 0.029), baseline IPSS (OR 1.3, p = 0.006), and SIL + Ser therapy (OR 1.76, p = 0.02) achieved independent predictor status for CMI#1, after accounting for Charlson Comorbidity Index (CCI), baseline maximal urinary flow rate (Qmax) and baseline prostate volume (PV). Similarly, younger age (OR 0.96, p = 0.023), baseline IPSS (OR 1.32, p = 0.003), and SIL + Ser therapy (OR 2.99, p = 0.011) were independently associated with CMI#2, after accounting for the same variables.

**Table 4:** Logistic regression models predicting clinically meaningful improvements in LUTS severity (CMI) (OR; *p* value [95% CI]) in the whole cohort.

	**CMI type # 1**		**CMI type # 2**	
	**UVA**	**MVA**	**UVA**	**MVA**
Age	0.96; 0.02	0.97; 0.029	0.97; 0.001	0.96; 0.023
	[0.95–0.98]	[0.87–0.96]	[0.97 – 0.99]	[0.91–0.99]
SIL vs. SIL + Ser	2.7; 0.001	1.76; 0.02	3.86; < 0.001	2.99; 0.011
	[1.46–4.6]	[0.63–1.9]	[2.2–6.71]	[1.22–7.11]
CCI ≥1	1.3; 0.93	2.51; 0.21	0.98; 0.72	0.49; 0.41
	[0.48–1.9]	[0.71–5.26]	[0.3–1.9]	[0.09–2.6]
Baseline IPSS	1.24; < 0.001	1.3; 0.006	1.45; 0.001	1.32; 0.03
	[1.1–1.3]	[1.1–1.32]	[1.02–1.6]	[1.06–1.32]
Baseline Flow max	0.9; 0.45	0.98; 0.19	0.96; 0.08	0.99; 0.35
	[1.0–1.2]	[0.97–1.15]	[0.92–1.2]	[0.93–1.09]
Baseline Prostate Volume	1.05; 0.09	1.1; 0.22	1.5; 0.23	1.1; 0.91
	[0.98–1.05]	[0.97–1.23]	[0.97–1.2]	[0.95–1.2]

Keys: UVA = Univariable analyses; MVA = Multivariable analyses; SIL = Silodosin; SIL + Ser = Silodosin + hexanic extract of serenoa repens; CCI = Charlson Comorbidity Index. IPSS = International Prostatic Index Score; CMI = Clinically meaningful improvements.

## Discussion

This cross-sectional study was designed to evaluate the prevalence and predictors of CMIs in LUTS severity in men with LUTS/BPH treated for ≥12 months with either SIL monotherapy or SIL + Ser as a combination therapy in the real-life setting. Clinically meaningful, patients treated with SIL + Ser reported greater improvement in terms of IPSS scores at follow-up assessment compared to men treated with SIL alone, with comparable rates of TEAEs. Likewise, the combination of SIL + Ser more frequently promoted CMIs achievement as compared with SIL alone.

Current EAU guidelines have not yet recommended the use of phytotherapeutic agents in the daily clinical practice because of several methodological biases in the published literature^1^. Furthermore, the heterogeneity of previous studies, the lack of adequately long-term follow-up and of any placebo arm, and especially the diverse types of extraction of SeR still limit the correct interpretation of the effectiveness of SeR itself and versus alpha-blockers^12^. In this context, our interest was fuelled by those existing controversies regarding the beneficial effect of phytotherapeutic agents^1,6–8^, either alone^8–12^ or in combination with alpha-blockers^13,14^.

Previous studies have shown that saw palmetto (320 mg) was effective in reducing prostate size and urinary frequency in patients with LUTS/BPH^15^. Moreover, numerous studies have shown that Ser has an efficacy equivalent to that of finasteride and tamsulosin for the treatment of LUTS/BPH, but with a reduced incidence of TEAEs^16,17^. Conversely, a systematic review reported that saw palmetto did not improve PV or urinary flow^6^. Likewise, previous studies reported that saw palmetto therapy was not superior to placebo in reducing LUTS, even at escalating doses^12,18^.

Only a few studies have investigated the benefit of the combination of Ser and alpha-blockers (any type), compared to alpha-blockers alone, in men with LUTS/BPH, with not unequivocal findings. A prospective, randomized trial compared the efficacy of Ser + tamsulosin vs. tamsulosin alone in a 12-mos treatment reporting that the combination was more effective than monotherapy in reducing storage symptoms in BPH patients^14^. In contrast, Argirovic found that the addition of Ser to tamsulosin did not lead to any clinical benefits in terms of IPSS score, Qmax or PVR^19^. Similarly, Hizli *et al*.^13^ evaluated the efficacy of Ser + tamsulosin vs. monotherapies and found no differences among groups in terms of IPSS and Qmax. However, these studies had several limitations, thus including the lack of detailed information about the type of extraction of Ser which may eventually impact on the quality of the compound.

For the specific purpose of this study, SIL has been chosen because its well-known selectivity and effectiveness in relieving BPH/LUTS^20,21^.

Our findings suggested that mean IPSS score was significantly lower for patients in the combination treatment group compared to those treated with SIL alone after a minimum of 12 months of daily therapy. A similar result was also observed in terms of both storage and voiding symptoms. Moreover, in contrast to other studies showing similar improvements in LUTS-related QoL in patients treated with alpha-blockers or Ser + alpha blockers^13^, we found that patients receiving SIL + Ser had a greater improvement in terms of IPSS-QoL than those treated with SIL alone.

Confirming previous studies, all patients showed improved Qmax with no statistical difference between treatment groups. Limited studies have evaluated PVR in measuring response to treatment^13,22^; in this context, we measured a comparable mean PVR decrease between groups.

As expected^23^, Ser showed no effects on PSA levels, thus confirming the high safety profile of the compound since decreasing PSA would not be a desired result of a BPH medication.

Of clinical relevance, no studies have investigated CMIs in LUTS severity in men treated with alpha-blockers or Ser + alpha-blockers. For instance, the PRECOMB trial compared the efficacy of Ser 320 mg combined with Selenium and Lycopene + tamsulosin 0.4 mg vs. the two monotherapies alone in men with LUTS/BPH^24^. They found that CMIs were more frequently reported in the group who had received the combination therapy as compared with those receiving the two treatments as a single regimen. Similarly, we found that a decrease of at least 3 points of the full IPSS score was achieved by 70% of SIL + Ser patients and a reduction of 25% of the same index was obtained in 69% of the combination group, thus showing significant advantages over SIL monotherapy for both outcomes (all p < 0.001). To this regard, younger age, baseline IPSS, and SIL + Ser therapy were independent predictors for both CMIs. Taken together, these findings would suggest that the addition of Ser 320 hexanic lipidosterolic extract to SIL may give rise to significant improvements of both IPSS scores and patient perception of LUTS severity (as assessed by CMIs), especially in young patients with severe symptoms. This sounds of clinical relevance since previous studies have reported that patients with BPH and prostate chronic inflammation not only have a higher risk of progression, but also lower rates of response to medical therapy^2–4^.

Several studies have confirmed the close correlation between the severity of LUTS and the prevalence of erectile dysfunction^25,26^. Our data showed no difference between groups regarding IIEF-EF scores at the mean follow-up assessment.

Of further clinical relevance, Ser 320 mg hexanic lipidosterolic extract was well tolerated by most users, and its daily intake was not associated with serious adverse events. Overall, 54 (28.2%) patients had at least one drug-related adverse event, with no significant differences between groups. As expected, ejaculatory disorders emerged as the most frequently reported AEs^5,20,27^; TEAEs did not result in withdrawal from the study in any case, thus confirming the high tolerability profile of both compounds in LUTS/BPH patients.

This real-life study is the first to assess CMIs in terms of LUTS severity in men treated with SIL alone or SIL + Ser. Major strength of the study was a rigorous methodology as compared to previous studies which demonstrated a number of methodological limitations and flaws^12^. Indeed, some studies were limited by a short follow-up (no longer than 6 months) and others failed to report precise data about the extraction modality about the Ser used^13,19^. In contrast, our study depicted strict inclusion criteria, with the goal to assess the efficacy of a defined type of Ser extract (namely, hexanic lipidosterolic extract) on LUTS severity after a homogenously relatively long follow-up period (i.e., ≥12 months).

Our study is not devoid of limitations. The study was retrospective and not randomized, but the matched-pair analysis significantly reduced the selection bias of a retrospective analysis; of relevance, patients data were stored prospectively; thereof, these findings certainly deserve external validation with an independent, larger and more diverse sample to be further confirmed. Moreover, although the baseline characteristics of the groups were comparable, the study lacked a placebo arm to further exclude any potential unknown confounding factors. Partially acquitting, we believe these findings are clinically relevant because of their robust characterization in the context of the real-life setting, since we decided to treat a large cohort of patients who consecutively had been seeking medical help for LUTS of a severity which actually deserved medical treatment according to EAU guidelines.

In conclusion, our cross-sectional, real-life study provides new clinically-relevant evidence that a combination therapy of SIL + Ser leads to greater clinically meaningful improvements in LUTS severity, compared to SIL as a monotherapy, after at least 12 months of treatment in men with moderate-to-severe LUTS/BPH. Both storage and voiding symptoms were significantly ameliorated by the combination therapy. Overall, keeping in mind the detrimental impact of LUTS/BPH on patients QoL, finding a therapeutic option which leads to greater clinically meaningful improvements in symptomatic terms along with a comparable rate of drug-related adverse events as compared with the most-widely first-line drugs clearly emerges as a major clinical need.

## Methods

Data from 190 Caucasian-European men seeking medical help for LUTS/BPH at a single academic outpatient clinic between January 2013 and September 2015 were analyzed. Data was prospectively collected and patients were retrospectively assigned to one of two groups based on the treatment they had undergone: SIL (8 mg once daily) alone or SIL + Ser (8 mg + 320 mg once daily) for no less than 12 months. Treatment allocation (i.e., a combination therapy vs. SIL alone) was defined according to the transrectal ultrasound (TRUS) findings indirectly suggestive or not for a prostate chronic inflammatory pattern.

Patients were retrospectively included in the study if they had an IPSS ≥12; a maximal urinary flow rate (Qmax) of ≤15 ml/s with a postvoiding residual volume (PVR) ≤150 ml; a prostate volume (PV) ≤90 cc (assessed by TRUS); serum prostate specific antigen (PSA) ≤4 ng/ml; and a digital rectal examination not suspicious for nodules at baseline. Exclusion criteria were the presence of a known prostate or bladder cancer; neurogenic disorders; a history of bladder disease or other urologic conditions likely to affect micturition (e.g. urethral stenosis, urinary incontinence, chronic bacterial prostatitis); symptomatic urinary tract infection; current anti-androgen or antidepressant therapy; previous treatment with any alpha-blockers (within 3 months), 5ARIs or other phytotherapeutic agents; patients with an indwelling catheter or with an episode of acute urinary retention over the last 4 weeks and previous surgical treatment for LUTS/BPH.

A detailed medical and sexual history was collected for every patient. Health-significant comorbidities were scored with the Charlson Comorbidity Index^28^ (CCI; categorized 0 vs. ≥1). Measured body mass index (BMI) was considered for each patient.

Patients completed the IPSS, with scores 1–7, 8–19, and 20–35 representing mild, moderate, and severe symptoms, respectively. Storage symptom scores (items 2, 4 and 7) and voiding symptom (items 1, 3, 5 and 6) scores were considered for each patient. Quality of life (QoL) question from IPSS was calculated as well. Patients also completed the IIEF-EF both at baseline and at follow-up assessment.

The assessment visit was performed in person after a minimum of 12 months. At follow-up, the IPSS, IIEF-EF, free uroflowmetry results, and PV were recorded. Similarly, TEAEs were also recorded. After treatment, clinically meaningful improvements in terms of LUTS severity were considered as: 1) a decrease of at least 3 points in the IPSS score (namely, CMI#1); and, 2) a decrease of 25% of the IPSS full score (namely, CMI#2)^29^.

The primary endpoint of the study was to assess the proportion of patients treated with combination therapy who achieved CMI#1 and CMI#2 as compared with those treated with SIL alone. We also evaluated the change from baseline to follow-up in terms of IPSS and IIEF-EF scores and in the efficacy parameters between groups.

Data collection was carried out following the principles outlined in the Declaration of Helsinki; after approval of the IRCCS San Raffaele Hospital’s Ethical Committee, all patients signed an informed consent agreeing to supply their own anonymous data for this and future studies.

Data are presented as means (SD; ranges). In order to control for measurable baseline differences among patients in the two treatment groups, adjustment was performed using 1:1 propensity-score matching^30^. After matching, 186 individuals were considered for the final analysis.

The statistical significance of differences in means and proportions was tested with the one-way analysis of variance (ANOVA) and Pearson chi-square test, respectively. A 95% confidence interval (95% CI) was estimated for the association of categorical parameters. Exploratory analyses were initially applied to all variables; variables were retained for analysis when deemed clinically significant to the results. Univariable and multivariable logistic regression analyses tested potential predictors of CMI in our cohort of men. Statistical analyses were performed using SPSS statistical software, v 13.0 (IBM Cor., Armonk, NY, USA). All tests were two sided, with a significance level set at 0.05.
